# Facteurs déterminants le regret après ligature tubaire

**DOI:** 10.11604/pamj.2014.17.244.4157

**Published:** 2014-04-01

**Authors:** Houssine Boufettal, Sakher Mahdaoui, Naïma Samouh

**Affiliations:** 1 Service de Gynécologie - Obstétrique « C », Centre Hospitalier Universitaire Ibn Rochd, Faculté de Médecine et de Pharmacie, Université Aïn Chok, Casablanca, Maroc

**Keywords:** Regret, ligature des trompes, stérilisation, contraception, Regret, tubal ligation, sterilization, contraception

## Abstract

**Introduction:**

La ligature tubaire peut générer regret. Le but de cette étude est d’étudier le vécu après la stérilisation ainsi que le regret et ses facteurs de risque.

**Méthodes:**

Il s'agit d'une étude rétrospective chez 52 femmes stérilisées entre 2004 et 2010.

**Résultats:**

Vingt cinq (48%) femmes avaient regretté la réalisation de la ligature tubaire. La moyenne d’âge était de 40,5 ans. Le temps consacré aux explications était très court dans tous les cas. Trois quart des femmes n’était pas au courant des complications de la ligature tubaire. Le regret était dû au facteur religieux (23%), aux algies pelviennes (11,5%), au désir d'autres enfants (9,6%) ou à la prise de connaissance d'autres moyens contraceptifs (3,9%).

**Conclusion:**

Un certains nombre de facteurs de risque de regret de la ligature tubaire sont retrouvés dans la littérature, comme dans notre série, dont l’âge, le désir de procréer, l'information incomplète, le délai de réflexion et le facteur religieux.

## Introduction

La ligature tubaire (LT) est une méthode de stérilisation permanente et irréversible. Il s'agit d'une intervention chirurgicale mineure qui consiste à obstruer les trompes de Fallope [1–5]. C'est l'un des moyens les plus efficaces de prévention des grossesses non désirées [6, 7]. Le choix d'une méthode de contraception classique ou de la ligature tubaire est très variable d'un pays ou d'une culture à l'autre, faisant intervenir de nombreux facteurs sociaux, économiques, religieux, culturels et personnels [8–16]. Malgré le développement des nouvelles technologies de la contraception, la stérilisation des trompes continuent de figurer parmi les méthodes, de limitation des naissances, les plus largement utilisés [17–25].

Bien qu′elle soit encore non régit par la loi au Maroc, la ligature tubaire a été pratiquée dans le secteur privé et le système de santé publique depuis de nombreuses années. Mais, ce geste, quoique simple techniquement, pose délicatement plusieurs problèmes [25]. Le moment de réalisation de ce geste pose difficulté, en effet, durant une intervention sur la cavité abdominopelvienne survenue d'une façon inattendue, et posant le choix d'une stérilisation tubaire, ne laisse pas assez de temps d'explications par le médecin, que ça soit sur la notion d'irréversibilité ou sur les effets secondaires, souvent négligés et ou mal connus, de cette méthodes, ni de réflexions pour le couple, ce qui est à l'origine de regret et d'un retentissement sur la qualité de vie et d'une souffrance psychologique [3–5]. L'absence de loi régissant la pratique de cette stérilisation tubaire, ouvre la porte à l'improvisation et parfois l'anarchie quand aux démarches administratives et médicales suivies pour avoir un consentement éclairé et volontaire de la patiente. Le but de notre travail est de déterminer les facteurs de risque de regret chez des patientes qui ont subit une ligature tubaire au Maroc et de comparer ces données avec celles de la littérature.

## Méthodes

Il s'agit d'une étude rétrospective ayant concerné 52 femmes de la population de Casablanca ayant subit une LT entre 2004 et 2010 dans le service de gynécologie-obstétrique « C » du Centre Hospitalier Universitaire Ibn Rochd de Casablanca. Les cas étaient contactés, soit par téléphone ou en se déplaçant à leur domicile. L’échantillon de l’étude était répartit en deux groupes: un groupe 1 (G1) de 25 femmes qui avaient regretté la ligature tubaire et un groupe 2 (G2) de 27 femmes qui n'avaient pas regretté cette ligature. La période de l'enquête était de 64 jours. L’étude était réalisée sous forme d'un questionnaire, grâce auquel nous avons pu recueillir des informations sur le vécu de la stérilisation tubaire. Le test Student était utilisé pour réaliser les calculs statistiques.

## Résultats

Parmi les 52 femmes qui avaient subit une LT ce groupe, 25 (48,1%) femmes avaient regretté la réalisation de la ligature tubaire. La moyenne d’âge était de 40,5 ans avec des extrêmes allant de 31 à 50 ans. Cette moyenne d’âge était de 38,7 ans chez les femmes qui avaient présenté un regret de la ligature tubaire et de 42,2 ans chez celles qui ne l'avaient pas présenté. La répartition selon l’âge est résumée dans le Tableau 1. L’âge inférieur à 39 ans était lié au regret de la ligature tubaire (OR 3.76). La moyenne d'enfants était de six. La répartition selon la parité est représentée dans le Tableau 2. Il n'existait pas de différence significative concernant la parité des deux groupes de femmes. Les antécédents médicaux comprenaient dans huit (15,4%) cas une cardiopathie à type de rétrécissement mitral séré avec hypertension artériel portale, dans six (11,5%) femmes un diabète non insulinodépendant, dans quatre (7,7%) une hypertension artérielle et dans deux (3,8%) cas une maladie de Crohn. L'interruption médicale de la grossesse était notée dans les antécédents de deux femmes, les fausses couches spontanées étaient retrouvées chez 10 (19,2%) cas, quatre (7,7%) femmes avaient l'antécédent d'une mort foetale in utero et deux femmes avaient un antécédent d'une hémorragie de la délivrance.

**Tableau 1:** Répartition selon les tranches d’âge

Tranche d’âge (en années)	G1 et G2 (N et%)	G1 (n et%)	G2 (n et%)	Odds Ratio
31 à 34	3 (5,8)	2 (3,9)	1 (1,9)	1.08
35 à 38	11 (21,1)	8 (15,4)	3 (5,8)	3.76
39 à 42	17 (32,7)	10 (19,2)	7 (13,5)	1.9
43 à 46	18 (34,6)	5 (9,6)	13 (25)	0.27
47 à 50	3 (5,8)	0	3 (5,7)	-
**Total**	52 (100)	25 (48,1)	27 (51,9)	**-**

G1 : groupe 1 de 25 femmes qui avaient regretté la ligature tubaire ; G2 : groupe 2 de 27 femmes qui n'avaient pas regretté la ligature

**Tableau 2:** Répartition selon la parité

Parité	G1 et G2 (N et%)	G1 (n et%)	G2 (n et%)	Odds Ratio
Quatre	8 (15,4)	6 (11,5)	2 (3,8)	3.95
Cinq	18 (34,6)	10 (19,2)	8 (15,4)	1.58
Six	15 (28,8)	6 (11,5)	9 (17,3)	0.63
Sept	9 (17,3)	3 (5,8)	6 (11,5)	0.48
Huit	2 (3,9)	0	2 (3,8)	-
**Total**	52 (100)	25 (48,1)	27 (51,9)	-

G1 : groupe 1 de 25 femmes qui avaient regretté la ligature tubaire ; G2 : groupe 2 de 27 femmes qui n'avaient pas regretté la ligature

Concernant les méthodes contraceptives utilisées par les femmes avant la ligature, la pilule était déjà utilisée par 42 (80,8%) femmes, 19 (36,5%) femmes avaient utilisé le dispositif intra-utérin, 29 femmes (55,8%) avaient utilisé l'abstinence et 12 (23,1%) femmes avaient utilisé le préservatif. L'intolérance à la pilule était une raison d'abondant chez 19 (36,5%) femmes, l'oubli l’était chez 12 (23,1%) femmes, deux femmes avaient fait une infection sur dispositif intra-utérin et 12 femmes (23,1%) qui utilisaient l'abstinence avaient pour raison l’échec de cette dernière. Les femmes qui étaient motivée pour la réalisation de la ligature tubaire étaient au nombre de 32 (61,5%). Les autres (38,5%) étaient non encore motivées au moment de la proposition de celle-ci. Les raisons de la motivation pour la ligature tubaire étaient représentées par l’âge avancé pour six (11,5%) femmes, la parité élevée pour six (11,5%) femmes (17%). La pathologie médicale associée était une raison motivant la ligature tubaire pour dix (19,2%) patientes, dont six cardiopathies et quatre diabétiques. Aussi, l'efficacité de la méthode était une raison annoncée par 12 femmes (23,1%). L'antécédent d'une hémorragie de la délivrance était une raison pour deux femmes (Tableau 3). La ligature tubaire était proposée par le médecin dans 22 (42,3%) cas, par la patiente dans deux cas et par les deux dans 28 (53,8%) cas.

**Tableau 3:** Raisons de la motivation des femmes pour la ligature tubaire

Les raisons de motivation	n	%
Efficacité	23	44,2
Age avancé	9	17,3
Nombre d'enfants	8	15,4
Cardiopathie	6	11,5
Diabète	4	7,7
Hémorragie délivrance	2	3,8
**Total**	52	100

La durée des explications données par le médecin à la femme sur la ligature tubaire était en moyenne de neuf minutes (une à 45 minutes). Ces données étaient expliquées entre une et cinq minutes pour 32 femmes (61,5%), entre six et dix minutes pour quatre femmes (7,7%), entre 11 et 15 minutes pour huit (15,4%) et entre 16 et 30 minutes pour cinq femmes (9,6%). Trois (5,8%) avaient reçu l'explication dans plus de 30 minutes (Tableau 4). La décision de la ligature tubaire était prise par la patiente seule dans huit cas (15,4%). Quatorze femmes (26,9%) l'avaient pris à la proposition du médecin, quatre femmes (7,7%) à l'aide du conjoint et 26 femmes (50%) à l'aide du médecin et du conjoint ensemble. Le délai de réflexion était compris entre une et dix minutes pour 14 (26,9%) femmes, 16 (30,8%) femmes avaient pris un délai entre 11 et 20 minutes. Chez quatre femmes (7,7%), ce délai était entre 21 et 30 minutes. Chez huit (15,4%) femmes, il était entre 31 et 120 minutes et chez deux femmes, ce délai était de plus de six mois. La période de réalisation de la ligature tubaire était au cours d'une césarienne chez 38 (73,1%) femmes. Quatre (7,7%) femmes l'avaient fait dans la période de moins de 45 jours après l'accouchement. Plus de 45 jours après l'accouchement étaient la période de ligature chez deux (3,8%) femmes. Huit (15,4%) femmes l'avaient réalisée au cours d'une intervention gynécologique (Tableau 5). Les femmes qui étaient au courant des complications de la ligature tubaire étaient au nombre de 12 (23,1%) et que 40 femmes (76,9%) n’étaient pas avertis quand aux effets secondaires de cette stérilisation. Aussi, les femmes qui étaient au courant de l'irréversibilité de la ligature tubaire représentaient 38 (73,1%) cas et 14 (26,9%) n’étaient pas au courant de l'irréversibilité de celle-ci. Les femmes qui avaient des effets secondaires après la ligature tubaire étaient au nombre de 22 (42,3%). La ligature tubaire était un bon choix, par son efficacité, pour 30 femmes (57,7%). Pour 22 (42,3%) femmes, cette ligature tubaire était un mauvais choix. La moitié des femmes avaient regretté la ligature tubaire. Les raisons du regret de la ligature tubaire étaient d'ordre religieux pour 14 (26,9%) femmes, les algies pelviennes chroniques pour six (11,5%) femmes, le désir d'avoir d'autres enfants pour huit (15,4%) femmes et l'existence d'autres moyens contraceptifs réversibles, non connus auparavant, pour deux femmes. Les raisons du non-regret étaient la présence d'une cardiopathie décompensée chez six (11,5%) femmes, de diabète compliquée chez quatre (7,7%) femmes et l'efficacité de la méthode chez 18 (34,6%) autres femmes (Tableau 6).

**Tableau 4:** Durée d'explication des données sur la ligature tubaire par le médecin

Durée (en minutes)	N	%
Entre 1 et 5	32	61,5
Entre 6 et 10	4	7,7
Entre 11 et 15	8	15,4
Entre 16 et 30	5	9,6
Plus de 30	3	5,8
**Total**	52	100

**Tableau 5:** Période de réalisation de la ligature tubaire

Période	N	%
Trans-césarienne	38	73,1
Au cours d'une intervention gynécologique	8	15,4
Moins de 45 jours après accouchement	4	7,7
Plus de 45 jours après accouchement	2	3,8
**Total**	52	100

**Tableau 6:** Raisons du regret de la ligature tubaire

Raisons	n	%
Religion	12	23,1
Algies pelviennes	6	11,5
Désir d'enfants	5	9,6
Autre moyen réversible	2	3,9
Pas de regret	27	51,9
**Total**	**52**	**100**

Deux femmes sur trois dans la tranche d’âge entre 31 et 34, avaient regretté la ligature tubaire (OR 1.08). Huit femmes parmi les 11 dans la tranche d’âge entre 35 et 38 ans avaient regretté la stérilisation (OR 3.76). Dix femmes parmi les 17 de la tranche d’âge comprise entre 39 et 42 ans avaient regretté la ligature tubaire (OR 1.9). Six femmes parmi les 18 dans la tranche d’âge de 43 à 46 ans avaient regretté le geste de ligature (OR 0.27) et aucune femme n'avait regretté le geste dans la tranche d’âge au-delà de 47 ans. Le regret de la ligature tubaire parait être lié à l’âge, plus la femme était jeune, plus le regret était possible. Le regret de la ligature tubaire était noté chez six femmes ayant une parité de quatre enfants (OR 3.95), chez dix femmes ayant cinq enfants (1.58), chez six femmes ayant une parité de six (OR 0.63) et chez trois femmes ayant une parité de sept enfants (OR 0.48). Aucun regret n’était noté avec une parité de huit. Ainsi, plus la parité augmente, plus le regret de la ligature tubaire diminue. Le désir d'avoir de nouveaux enfants après la ligature tubaire était présent chez dix (19,2%) femmes. Deux femmes avaient perdu le dernier enfant dans la petite enfance et deux avait le désir d'avoir un enfant de sexe différent. Six ne donnaient pas d'arguments précis. Les femmes qui pouvaient recommander la ligature tubaire à une autre proche ou amie étaient au nombre de 24 (46,2%). Par contre, 28 cas (53,8%) disaient qu'elles ne la recommanderaient pas à une autre femme.

## Discussion

Le regret de la LT se définit par le fait de changer d'avis après la réalisation de celle-ci [1–5]. La fréquence de ce regret est diversement variable dans la littérature. Elle varie de 3 à 26% [5–13, 15–18]. Dans une vaste étude prospective multicentrique réalisée aux Etats-Unis portant sur 5817 patientes interrogées trois à cinq ans après leur stérilisation, Wilcox et al. [13] mettent en évidence deux fois plus de regrets chez les patientes stérilisées avant l’âge de 30 ans. Dans une étude américaine, Rosenfeld et al. [2] retrouvent 76,8% de femmes stérilisées avant l’âge de 30 ans parmi le 21,9% de patentes regrettant leur intervention. Ceci n'est pas le cas dans notre étude puisque l’âge à partir duquel la ligature tubaire était faite est de 33 ans. Notre étude montre un taux du regret très grand par rapport aux autres études, et ceci, pourrait être expliqué, par deux raisons principales, d'une part, l'indication faite à la hâte, généralement en trans-césarienne (73,1%), et d'autre part, par le facteur religieux (23,1%), qui interdisait les modes de contraception irréversible, notion qui n’était pas connue par les femmes, de cette enquête, avant la réalisation de la ligature tubaire.

Plusieurs études rapportent une moyenne d’âge de la ligature tubaire entre 35 et 40 ans et qu'au-delà de cette tranche d’âge, le taux de stérilisation devient de plus en plus important (3,9% à 35-39 ans, 6% à 40-44 ans et 8,4% à 45-49 ans) [1–5]. La moyenne d’âge dans notre étude était de 40,5 ans. En revanche, cette moyenne d’âge était plus basse (38,7 ans) chez les femmes qui avaient présenté un regret de la ligature tubaire par rapport à celles qui ne l'avaient pas présenté (42,2 ans). Ainsi, l’âge parait un facteur de risque de regret de la ligature tubaire, notamment pour des tranches inférieures à 39 ans. Ce sont les femmes ayant un nombre suffisant et varié d'enfants qui ont le plus souvent recours à la ligature tubaire [12, 24, 25]. En effet, la moyenne de parité, dans notre étude, est de six enfants. Le regret de ligature tubaire était plus fréquent pour une parité de quatre (OR 3.95). Pour une parité de six ou plus, le taux de regret est plus faible (OR 0.63 à 0.48). La grossesse est un moment délicat chez la patiente porteuse d′une maladie chronique (cardiopathie, diabète, hypertension artérielle, lupus, etc) par le risque important d'accidents gravido-cardiaques (oe'dèmes, avortements, accouchements prématurés, etc.). Les suites de couches posent le problème des thromboses veineuses (phlébite, oe'dème aigu du poumon). Dans notre étude, huit (15,4%) femmes présentaient une cardiopathie à type de rétrécissement mitrale avec hypertension artérielle pulmonaire, suivi du diabète chez six (11,5%) cas et l'hypertension artérielle chez quatre (3,8%) cas. Il n'existait pas de différence significative en matière de regret chez les femmes qui présentaient ces pathologies chroniques. Ainsi, la présence de telles pathologies parait protectrice vis-à-vis du regret de la ligature tubaire. L'oubli et l'intolérance de la pilule sont les principales raisons d'abandon de cette dernière. En effet, jusqu’à 60% des femmes indiquent avoir oublié leur pilule au moins une fois [19]. L'abstinence périodique, même bien maîtrisée, présente un taux d’échec d'environ 20% [4–6]. En raison de ses effets secondaires, ses contre-indications chez les femmes ayant un terrain à risque vasculaire, ainsi que son taux d’échec qui n'est pas minime, notamment avec la mauvaise observance et compliance, la pilule risque d’être abandonnée [19]. Ainsi, dans notre étude, l'intolérance à la pilule et l’échec de l'abstinence étaient les premières raisons d'abandon de cette méthode contraceptive chez respectivement, 19 (36,5%) et 12 (23,1%) des femmes, suivies de l'oubli de la pilule chez 12 (23,1%) femmes. L'infection sur dispositif intra-utérin était notée chez deux femmes. Aussi, dans une étude effectuée en 2000 sur 168 patientes [8], les raisons de choix de la ligature tubaire étaient dominées par les effets secondaires de la méthode contraception antérieure et le souhait de ne plus avoir à se préoccuper du sujet de la contraception, alors que 2% des cas présentaient une contre-indication à la grossesse. Dans une étude effectuée entre 1975 et 1985 par Burnel et al. [3], les motivations pour la stérilisation tubaire étaient le nombre d'enfants désirés atteint ou dépassés pour 45,5% des cas, l’âge élevé de la femme dans 20,4% des cas. Une étude menée sur des femmes afro-américaines et blanches en 2008 par Borrero et al. [17] a révélé que des raisons de motivation des femmes pour la ligature tubaire représentées par la taille de la famille, l’âge avancé et les difficultés liées à l’éducation des enfants, la prévention de futures grossesses non désirées, en particulier chez les femmes célibataires. Dans notre étude, les femmes motivées pour la ligature tubaire avaient pour raisons en premier lieu l'efficacité de la ligature tubaire chez le tiers d'entre elles. Chez les autres, les raisons étaient représentées par l’âge avancé, la parité élevée et la présence d'une pathologie contre-indiquant la grossesse.

La demande de la LT parvenait à la fois du médecin et de la femme dans 14 (26,9%) cas. Cette demande parvenait du médecin seul pour 14 (26,9%) femmes et seules huit (15,4%) femmes l'avaient elles mêmes demandées à leur médecin. La quasi-majorité des demandes des ligatures tubaires étaient au cours d'une césarienne. La durée de décision et d'explications concernant la ligature tubaire était moins de 30 minutes chez 38 (73,1%) femmes, ce qui est une durée insuffisante. Deux femmes seulement avaient eu une durée d'explication de plus de six mois. De ce fait, les connaissances des femmes à propos de la ligature tubaire étaient limitées. Seulement le quart des femmes étaient au courant des complications postopératoires. Or, les personnes demandeuses d′une stérilisation doivent faire l′objet d′une consultation médicale initiale, au cours de laquelle la personne sera informée des risques médicaux qu′elle encourt et des conséquences de l′intervention, et un dossier d′information écrite (servant de support à l′information orale) lui sera remis [5, 9]. Aussi, les femmes doivent disposer d′un délai de réflexion de quatre mois après la consultation médicale initiale, avant de confirmer leur volonté, par écrit [14]. Tout médecin sollicité par une personne demandeuse d′une stérilisation dispose d′une clause de conscience, mais doit l′informer de son refus dés la consultation initiale. Beaucoup d’études rapportent un taux de réalisation de la LT important (30 à 51%) en période trans-césarienne ou dans le post partum après un accouchement par voie basse et qui étaient associées à un risque élevé de regret [4, 21, 24]. Aussi, dans une population de 242 femmes nord-américaines stérilisées dans la période du post partum, Rosenfeld et al. [2] notent jusqu’à 21,9% de regret à cinq ans. Des rapports précédents ont identifié la stérilisation post-partum comme un facteur de risque de regret augmenté [6, 7, 11–13, 17]. Ainsi, la période de réalisation de la ligature tubaire peut constituer un facteur de risque de regret. En effet, au cours de césarienne ou dans le post-partum immédiat, la décision de stérilisation peut être prise à la hâte, dans les suites d'une grossesse ou d'un accouchement parfois pénibles, sous le poids des charges multiples d'une nouvelle maternité, à un moment ou existe toujours une incertitude sur l’état de santé du nouveau-né [17, 25]. La période de réalisation de la ligature tubaire des femmes de notre étude était pour la plupart (73%) en trans-césarienne. Les effets secondaires d'une stérilisation tubaire peuvent être aussi une raison de regret de celle-ci [20, 25]. Les femmes de l’étude qui avaient présenté des effets secondaires suite à la ligature tubaire représentaient 42% essentiellement à type d'algies pelviennes et ceci était parmi les raisons de regret de la ligature tubaire chez six (11,5%) femmes.

D'autres facteurs sont associés avec le regret de la ligature tubaire. Il s'agit du jeune âge et d'un imprévisible événement de la vie, tel que le changement de statut matrimonial, la mort d′un enfant, le désir d′enfant de sexe différent, le souhait de retrouver la fécondité, ou encore, des facteurs religieux et psychologique [6, 7, 11, 17]. Dans notre étude, l’âge jeune inférieur à 39 ans, le changement du statut matrimonial, le désir d'autres enfants, même chez des multipares, et les facteurs religieux et psychologiques étaient les principaux facteurs à l'origine du regret de la ligature tubaire. Contrairement à une idée encore trop largement répondu, la grande multiparité n'est pas un facteur réduisant l'incidence des regrets. Dans notre étude, neuf (17,3%) femmes ayant regretté avaient six ou sept enfants. Ceci est le cas, aussi, pour une étude effectué en Belgique en 2000 [8], où 58% des patientes avaient trois ou plus de trois enfants au moment de la stérilisation et parmi les 10% des patientes nullipares aucune n'a entrepris une démarche en vue d'une nouvelle grossesse. Plusieurs études ne retrouvent toutefois pas ce facteur de risque [13, 25]. Ainsi, il faut éviter une stérilisation chez une femme dont la décision n’était pas prise définitivement, qui hésite. Il faut être particulièrement prudent chez les femmes de moins de 35 ans, pour lesquelles le risque de regret est non négligeable [5, 25]. Les aléas de la vie doivent être évoqués avec les patientes et surtout celles dont la vie en couple n'est pas stabilisée.

## Conclusion

La LT est encore, souvent pratiquée, dans notre contexte, avec beaucoup de manque d'information et de motivation, des délais de réflexion courts et où le facteur religieux et le caractère irréversible de la technique sont des facteurs, non pris en compte. Pour minimiser le taux de regret après la LT, la femme doit être informée de tous les facteurs de risque de regret de celle-ci. Le délai de réflexion doit être de quatre à six mois avec notamment un manuscrit d'accord pour exprimer le consentement de la patiente au cours de la deuxième consultation. La stérilisation tubaire peut être recommandée comme une méthode sûre et efficace pour les femmes qui veulent une contraception permanente et ou porteuse d'une pathologie contre indiquant formellement la grossesse, à condition que cette maladie n'aura pas la possibilité d’être traitée ou guérie. Certes, si on évite à la femme de regretter la ligature tubaire, on ne lui évite pas certainement le regret de la grossesse. Le refus de la contraception permanente devrait amener à envisager d'autres méthodes de contraception « non eugéniques », qui ne sont peut être pas aussi sûres pour éviter le regret de la grossesse non désirée, mais, réversibles et permettent, sans doute d’éviter le regret de la ligature tubaire, ainsi que le vécu eugénique de celle-ci.
